# Temporal trends and disparities in mortality among US adults with chronic kidney disease and comorbid depression: a population-based analysis, 1999–2023

**DOI:** 10.3389/fpubh.2026.1860838

**Published:** 2026-06-03

**Authors:** Pengcheng Zhao, Libo Yang, Fang Wu, Yingan Li, Yun He

**Affiliations:** 1 Key Laboratory of Birth Defects and Related Diseases of Women and Children, Ministry of Education, West China Second Hospital, Sichuan University, Chengdu, Sichuan, China; 2 Department of Pediatric General Surgery, West China Second Hospital, Sichuan University, Chengdu, Sichuan, China

**Keywords:** CDC WONDER, chronic kidney disease, depression, health disparities, mortality trends

## Abstract

**Background:**

Chronic kidney disease (CKD) and depression frequently co-occur, yet population-level mortality trends for their joint occurrence are poorly characterized.

**Methods:**

Using the CDC WONDER multiple-cause-of-death database, we examined annual age-adjusted mortality rates (AAMR) per 100,000 population among U.S. adults aged ≥ 25 years with CKD and co-occurring depression from 1999 to 2023. Joinpoint regression was employed to estimate annual percent change (APC) and average annual percent change (AAPC). Trends were stratified by sex, age, race, census region, and urban–rural residence. A Poisson log-linear model tested trend heterogeneity between CKD overall and CKD with depression during 2016–2021.

**Results:**

11,076 deaths were attributed to CKD with co-occurring depression. Overall AAMR increased from 0.12 to 0.22 per 100,000 (AAPC, 2.32%; 95% CI, −3.48 to 8.47%). Joinpoint analysis identified a critical inflection point in 2015, after which mortality surged at an APC of 9.89% (95% CI, 6.39 to 13.50%; *p* < 0.001). In contrast, CKD overall mortality decelerated after 2016 and plateaued recently. The post-2015 slope for CKD with depression significantly exceeded that of CKD overall (*p* for interaction < 0.0001). Acceleration was most pronounced among men, adults aged ≥85 years, residents of the South, and nonmetropolitan populations. Sensitivity analysis restricted to 2013–2023 yielded a sustained upward APC of 7.38% (95% CI, 5.00–10.41%; *p* < 0.0001).

**Conclusion:**

Mortality among CKD with co-occurring depression has accelerated sharply since 2015, diverging markedly from the plateauing trend of CKD overall. Routine depression screening integration into nephrology care is urgently warranted, especially for high-risk populations.

## 1 Introduction

Chronic kidney disease (CKD) is a global public health concern, characterized by a progressive and irreversible decline in renal function. Its prevalence and incidence are increasing worldwide, affecting approximately 700 million adults (1, 2), posing a substantial burden on healthcare systems and exacting a profound toll on patients’ physical functioning and quality of life (3, 4). As CKD progresses, patients often experience a range of physical complications, including cardiovascular diseases (CVD), metabolic disorders, and mental health disorders (5). Among these complications, depression is a particularly prevalent and debilitating comorbidity, affecting an estimated 20–40% of CKD patients (6, 7). Depression in CKD patients is associated with a multitude of adverse outcomes, including reduced adherence to treatment regimens, increased risk of cardiovascular events, and accelerated progression of kidney disease, leading to a poorer prognosis (8).

The intricate relationship between CKD and depression is thought to be bidirectional. CKD-related physical symptoms, such as fatigue, pain, and fluid retention, can contribute to the development of depressive symptoms (9). Conversely, depression can exacerbate CKD progression by reducing dialysis adherence, diminishing therapeutic efficacy, increasing the risk of complications, and impairing health-related quality of life (10–13), ultimately contributing to adverse outcomes. Although previous studies have established an association between CKD and depression, most studies have investigated the risk factors for depression in CKD patients (14–17), or only focused on the negative impact of depressive symptoms on the outcome of CKD (18, 19). Given the high prevalence and detrimental consequences of depression in CKD, understanding its impact on mortality is of paramount importance. One study of Zhao et al. has evaluated depression levels in patients with CKD and examined their association with all-cause mortality using National Health and Nutrition Examination Survey (NHANES) merged with the National Death Index (NDI) data (20). They found a dose–response relationship, with higher depression levels corresponding to greater mortality risk. However, the temporal trends in mortality among individuals with CKD and depression at a national level have not been fully examined.

To address these knowledge gaps, this study aims to leverage the comprehensive mortality data available in the Centers for Disease Control and Prevention (CDC) Wide-ranging Online Data for Epidemiologic Research (WONDER) database to investigate the mortality trends and burden associated with CKD comorbid with depression in the United States and elucidate the relative contribution of various demographic, socioeconomic, and clinical factors to the excess mortality. Our findings are intended to identify vulnerable subpopulations, and provide an empirical foundation for urgently integrating mental health care into the mainstream management of chronic kidney disease.

## 2 Methods

### 2.1 Data source and study population

This study utilized publicly available mortality data from the Centers for Disease Control and Prevention (CDC) Wide-ranging Online Data for Epidemiologic Research (WONDER) database to examine mortality trends among U.S. adults with chronic kidney disease (CKD) and comorbid depression. The analysis was based on the Multiple Cause of Death Public Use files, encompassing data from all 50 states and the District of Columbia. The study population included U.S. decedents aged 25 years or older. CKD was defined based on the presence of ICD-10 codes N18 (Chronic Kidney Disease) listed as either the underlying or contributing cause of death on the death certificate. Depression was identified through ICD-10 codes F32 (Major depressive disorder, single episode) or F33 (Major depressive disorder, recurrent), documented in any of the multiple cause of death fields. Individuals meeting both the CKD and depression criteria were included in the primary study cohort. For comparative analyses, a separate cohort of decedents with CKD overall (with or without comorbid depression) was also extracted using the same ICD-10 code definition.

### 2.2 Data extraction

The following variables were extracted from the CDC WONDER database: year of death, age, sex, race/ethnicity, census region, state of residence, and cause of death codes. Age was categorized into the following 10-year intervals: 25–34, 35–44, 45–54, 55–64, 65–74, 75–84, and ≥85 years. Race/ethnicity was classified as Hispanic or Latino, Non-Hispanic (NH) White, NH Black or African American, and NH Other (including Asian, Native American, and other racial/ethnic groups). Geographic regions were defined according to US Census Bureau designations: Northeast, Midwest, South, and West. Urban–rural classification was based on the National Center for Health Statistics Urban–Rural Classification Scheme for Counties, dichotomized into metropolitan and nonmetropolitan categories. Notably, at the time of analysis, CDC WONDER had not released urban–rural mortality data for CKD with depression beyond 2020. For metropolitan and nonmetropolitan strata, the most recent available AAMR data (2020) were therefore used as the end point for trend analysis, and AAPCs for these strata were calculated over the 1999–2020 period.

### 2.3 Ethical considerations

This study involved secondary analysis of publicly available, de-identified human mortality data from the CDC WONDER database. Because the data are de-identified and publicly accessible, the study did not constitute human subjects research and was exempt from institutional review board (IRB) review. The study was conducted in accordance with the Strengthening the Reporting of Observational Studies in Epidemiology (STROBE) guidelines (21).

### 2.4 Statistical analysis

Crude mortality rates (CMR) and age-adjusted mortality rates (AAMR) per 100,000 population were calculated annually from 1999 to 2023 for patients with CKD and comorbid depression. AAMRs were calculated using the direct method with the 2,000 U.S. Standard Million Population as the reference. Because AAMRs are standardized to the general population, the reported rates reflect the population-level mortality burden of CKD with co-occurring depression rather than the case fatality rate among individuals with CKD. Confidence intervals (95% CI) for CMR and AAMR were calculated using the Poisson distribution. Rates were stratified by sex, race and ethnicity, age group, census region, and urban–rural residence.

Joinpoint regression analysis (Joinpoint software, Version 5.1.0.0; National Cancer Institute) was used to examine temporal trends in AAMRs. The analysis was performed with a maximum of three joinpoints allowed per model. The optimal number of joinpoints was selected using the permutation test with 4,499 random permutations and an overall significance level of α = 0.05. The minimum segment length was set to three data points to ensure interpretable segments. This method identifies statistically significant changes in the slope of the mortality trend over time, allowing for the calculation of APC and AAPC with 95% CIs (Supplementary Table S4). Parallelism tests were conducted to assess whether mortality trends differed significantly across demographic and subgroups. To compare mortality trajectories between CKD overall and CKD with co-occurring depression, annual AAMRs for both groups were plotted and analyzed using Joinpoint regression, and a Poisson log-linear regression model with a year-by-group interaction term was fitted to the 2016–2021 interval to formally test for trend heterogeneity. Overdispersion was assessed by comparing the residual deviance with the residual degrees of freedom. All statistical analyses were performed using R Statistical Software (version 4.4.1; R Foundation for Statistical Computing). A two-sided *p*-value < 0.05 was considered statistically significant.

## 3 Results

### 3.1 Overall

Between 1999 and 2023, a total of 11,076 deaths were attributed to CKD with co-occurring depression in the United States, of which 4,516 occurred in males and 6,566 in females. The AAMR increased from 0.12 (95% CI: 0.10 to 0.13) per 100,000 in 1999 to 0.22 (95% CI: 0.21 to 0.24) in 2023, representing a percent change of +200.96% over the study period and corresponding to an AAPC of 2.32% (95% CI: −3.48 to 8.47) (Table 1).

**Table 1:** CKD combined with depression deaths and AAMR in the United States from 1999 to 2023 and their changing trends.

Demographics	Deaths			AAMR (per 100,000)		
	1999	2023	Percent change (%)	1999 (95% CI)	2023 (95% CI)	AAPC (95% CI)
Total	209	629	200.96	0.12 (0.10 to 0.13)	0.22 (0.21 to 0.24)	2.32 (−3.48 to 8.47)
Sex	120	386	221.67	0.10 (0.08 to 0.12)	0.24 (0.21 to 0.26)	4.00 (−5.76 to 14.77)
Male	89	243	173.03	0.14 (0.11 to 0.18)	0.20 (0.18 to 0.23)	2.01 (−7.59 to 12.61)
Female	77	151	96.10	0.17 (0.13 to 0.21)	0.24 (0.20 to 0.28)	0.52 (−10.88 to 13.39)
Census region	40	108	170.00	0.10 (0.07 to 0.13)	0.20 (0.16 to 0.24)	1.11 (−8.77 to 12.06)
Northeast	61	225	268.85	0.09 (0.07 to 0.12)	0.23 (0.20 to 0.27)	4.00 (−5.86 to 14.88)
Midwest	31	145	367.74	0.08 (0.05 to 0.12)	0.24 (0.20 to 0.27)	4.06 (−2.05 to 10.55)
South	NA	29	NA	NA	0.11 (0.07 to 0.16)	NA
West	16	30	87.50	NA (0.06 to 0.17)	0.12 (0.08 to 0.18)	−2.75 (−15.05 to 11.32)
Race	182	555	204.95	0.13 (0.11 to 0.15)	0.28 (0.25 to 0.30)	2.81 (−2.61 to 8.53)
Hispanic	NA	15	NA	NA	NA (0.04 to 0.12)	NA
NH Black	209	629	200.96	0.12 (0.10 to 0.13)	0.22 (0.21 to 0.24)	2.32 (−3.48 to 8.47)
NH White	120	386	221.67	0.10 (0.08 to 0.12)	0.24 (0.21 to 0.26)	4.00 (−5.76 to 14.77)
NH Other	89	243	173.03	0.14 (0.11 to 0.18)	0.20 (0.18 to 0.23)	2.01 (−7.59 to 12.61)
Urbanization						
Metropolitan	155	566^a^	265.16	0.10 (0.08 to 0.11)	0.24 (0.22 to 0.26)^a^	3.78 (−1.74 to 9.60)^a^
Nonmetropolitan	54	137^a^	153.70	0.17 (0.12 to 0.22)	0.30 (0.25 to 0.35)^a^	2.68 (−5.80 to 11.91)^a^
Age groups^b^						
25–54 years^c^	NA	NA	NA	NA	NA	NA
55–64 years	13	37	184.62	NA (0.03 to 0.09)	0.09 (0.06 to 0.12)	1.24 (−11.43 to 15.74)
65–74 years	36	100	177.78	0.20 (0.14 to 0.27)	0.29 (0.23 to 0.34)	0.65 (−0.93 to 2.25)
75–84 years	74	177	139.19	0.61 (0.48 to 0.76)	0.96 (0.82 to 1.11)	2.53 (−7.53 to 13.68)
85+ years	72	286	297.22	1.73 (1.36 to 2.18)	4.62 (4.08 to 5.15)	4.29 (−3.98 to 13.26)

NA, not available; AAMR, age-adjusted mortality rates; AAPC, average annual percentage change; CI, confidential interval; NH, non-Hispanic.

a The AAMR data for 2023 was substituted with the 2020 data, while the AAPC was calculated over the period from 1999 to 2020.

b Crude rates were used instead of AAMR.

c The 25–54 years age group is too small in number.

NA appears: (1) death counts suppressed by CDC WONDER when fewer than 10 deaths occurred; (2) rates considered unreliable due to sparse data yielding wide or unstable confidence intervals; and (3) the 25–54 years age group, which had insufficient death counts throughout the study period and was excluded from analysis.

**Figure 1:**
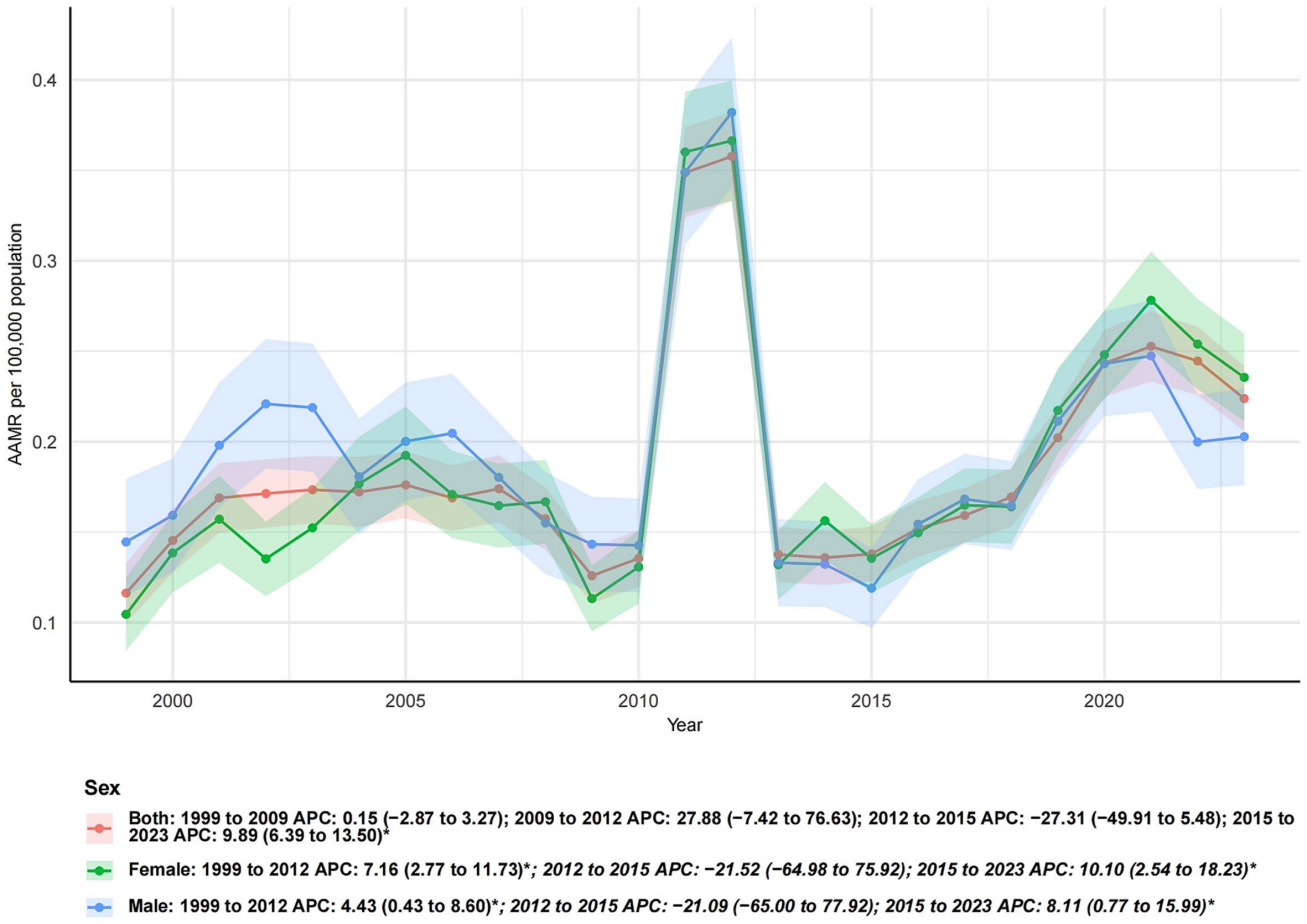
Twenty-five-year trends in mortality among CKD patients with depression by sex in the United States, 1999–2023. Annual percent changes (APCs) with 95% confidence intervals are shown for each demographic group across distinct time periods identified by joinpoint regression analysis. Asterisks (*) indicate statistically significant trends: *p < 0.05, Shaded areas represent 95% confidence intervals around the trend lines. AAMR, age-adjusted mortality rates; APC, annual percent change; CI, confidence interval.

Joinpoint regression identified four distinct phases in overall trends (Figure 1). From 1999 to 2009, the AAMR remained essentially stable, with an APC of +0.15% (95% CI, −2.87 to 3.27%; *p* = 0.916). This was followed by a non-significant surge between 2009 and 2012 (APC, +27.88%; 95% CI, −7.42 to 76.63%; *p* = 0.125), and a subsequent non-significant decline from 2012 to 2015 (APC, −27.31%; 95% CI, −49.91 to 5.48%; *p* = 0.087). In contrast, the most recent period (2015–2023) showed a significant and sustained increase, with an APC of +9.89% (95% CI, 6.39 to 13.50%; *p* < 0.001).

### 3.2 Sex-stratified analysis

Sex-specific AAMR trajectories exhibited a crossover pattern over the study period. In 1999, men had a higher AAMR (0.14 per 100,000; 95% CI, 0.11 to 0.18) than women (0.10 per 100,000; 95% CI, 0.08 to 0.12). However, by 2023, women’s AAMR (0.24 per 100,000; 95% CI, 0.21 to 0.26) had surpassed that of men (0.20 per 100,000; 95% CI, 0.18 to 0.23), reflecting a steeper overall increase among women (Table 1).

**Figure 2:**
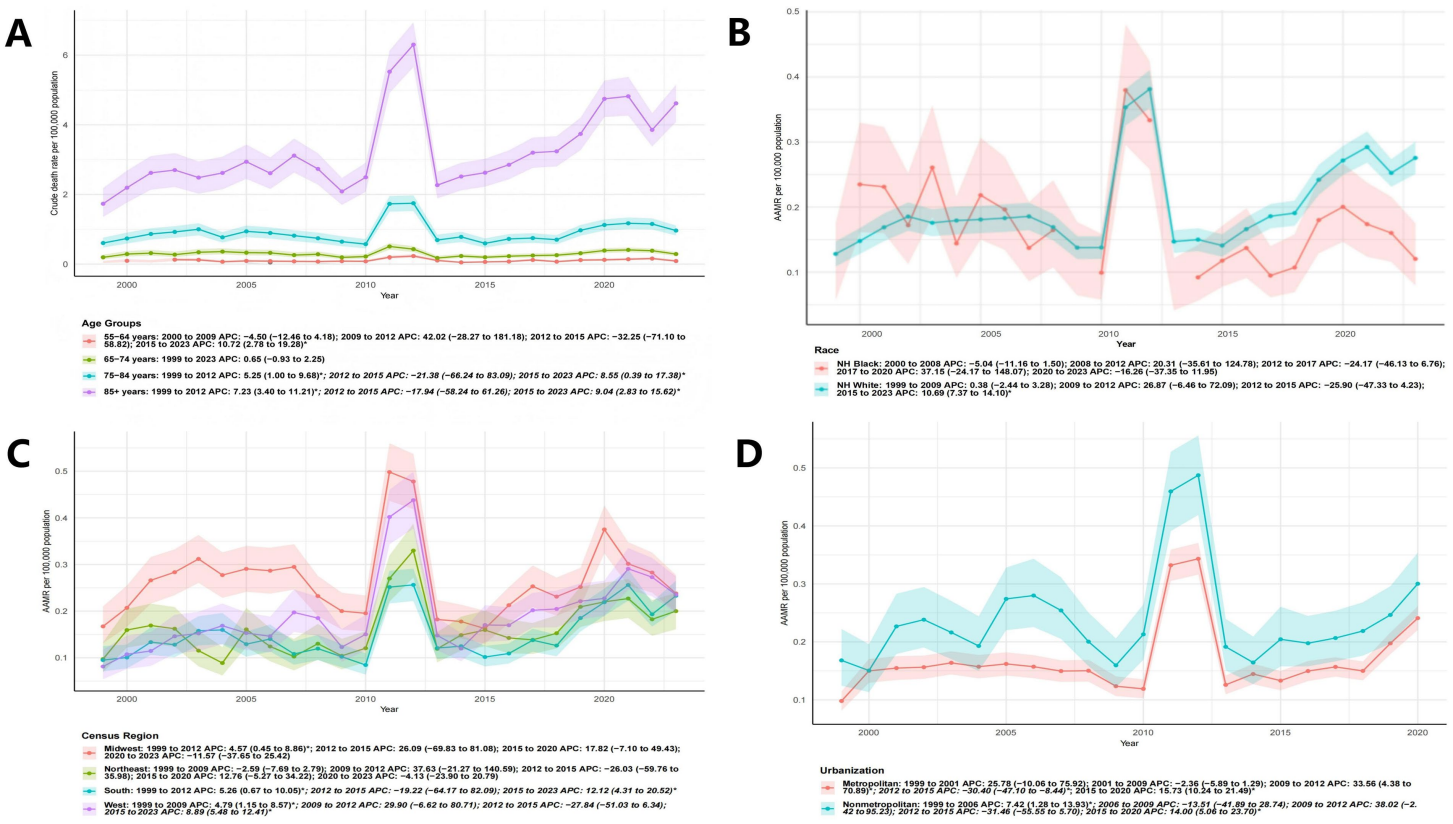
Annual trends in mortality among CKD patients with depression stratified by **(A)** age groups, **(B)** race/ethnicity, **(C)** census regions, **(D)** urbanization in the United States, 1999–2023. Annual percent changes (APCs) with 95% confidence intervals are shown for each demographic group across distinct time periods identified by joinpoint regression analysis. Asterisks (*) indicate statistically significant trends: *p < 0.05. Shaded areas represent 95% confidence intervals around the trend lines. AAMR, age-adjusted mortality rates; APC, annual percent change; CI, confidence interval; NH, non-Hispanic.

Despite this similarity in overall direction, joinpoint regression revealed distinct temporal patterns by sex. Among women, three distinct phases were identified: an initial increase from 1999 to 2012 (APC, +7.16%; 95% CI, 2.77 to 11.73%), followed by a non-significant decline between 2012 and 2015 (APC, −21.52%; 95% CI, −64.98 to 75.92%), and a significant acceleration from 2015 to 2023 (APC, +10.10%; 95% CI, 2.54 to 18.23%). Among men, a comparable three-phase pattern was observed: an increase during 1999–2012 (APC, +4.43%; 95% CI, 0.43 to 8.60%), a non-significant decline from 2012 to 2015 (APC, −21.09%; 95% CI, −65.00 to 77.92%), and a significant rise in the final segment (2015–2023; APC, +8.11%; 95% CI, 0.77 to 15.99%) (Figure 2A).

### 3.3 Age group-stratified analysis

Age-specific trends were assessed using crude mortality rates (CMR). Due to small numbers, data for adults aged 25–54 years were largely suppressed and excluded from trend analysis.

Adults aged 55–64 years showed an overall increasing pattern, with fluctuating segments: a decline during 2000–2009 (APC: −4.50%; 95% CI: −12.46 to +4.18%), a sharp but imprecise rise in 2009–2012 (APC: +42.02%; 95% CI: −28.27 to +181.18%), a nonsignificant decline in 2012–2015 (APC: −32.25%; 95% CI: −71.10 to +58.82%), and a significant increase from 2015 to 2023 (APC: +10.72%; 95% CI: +2.78% to +19.28%). Correspondingly, the 55–64 group experienced growth in the absolute number of deaths over the period, consistent with a positive AAPC and percent change in Table 1.

For adults aged 65–74 years, the overall trend remained relatively flat throughout the study period (APC for 1999–2023, +0.65%; 95% CI, −0.93 to 2.25%). Modest changes in CMR and death counts were observed, as reflected by the narrow range of the AAPC estimate (Table 1). The 75–84 years group exhibited a biphasic pattern: a significant increase from 1999 to 2012 (APC: +5.25%; 95% CI: +1.00% to +9.68%), followed by a decline in 2012–2015 (APC: −21.38%; 95% CI: −66.24% to +83.09%) and a subsequent significant rise from 2015 to 2023 (APC: +8.55%; 95% CI: +0.39% to +17.38%). Consistent with these segments, CMR and deaths in this age group rose overall, with positive AAPC and percent change. The oldest group (≥85 years) showed the highest levels and marked temporal variability. Joinpoint analysis identified an initial significant increase from 1999 to 2012 (APC, +7.23%; 95% CI, 3.40 to 11.21%), a non-significant decline between 2012 and 2015 (APC, −17.94%; 95% CI, −58.24 to 61.26%), and a significant increase from 2015 to 2023 (APC, +9.04%; 95% CI, 2.83 to 15.62%). Deaths in this group rose substantially from 72 in 1999 to 286 in 2023, contributing to the positive AAPC of 4.29% (95% CI, −3.98 to 13.26%) (Figure 2A).

### 3.4 Race-stratified analysis

Racial patterns were evaluated using AAMR. Among non-Hispanic (NH) White adults, joinpoint regression identified a multiphasic trajectory with a pronounced acceleration in recent years. From 1999 to 2009, rates were essentially stable (APC: +0.38%; 95% CI: −2.44% to +3.28%). This was followed by a brief, non-significant surge between 2009 and 2012 (APC, +26.87%; 95% CI, −6.46 to 72.09%), after which a decline emerged in 2012–2015 (APC: −25.90%; 95% CI: −47.33% to +4.23%). Subsequently, a sustained and significant increase was observed from 2015 to 2023 (APC: +10.69%; 95% CI: +7.37% to +14.10%). In contrast, NH Black adults exhibited greater volatility, with alternating periods of decline and increase. A non-significant decrease was observed from 2000 to 2008 (APC, −5.04%; 95% CI, −11.16 to 1.50%), followed by a short, statistically imprecise rise during 2008–2012 (APC, +20.31%; 95% CI, −35.61 to 124.78%). Rates then declined again between 2012 and 2017 (APC, −24.17%; 95% CI, −46.13 to 6.76%), rose sharply but non-significantly from 2017 to 2020 (APC, +37.15%; 95% CI, −24.17 to 148.07%), and decreased once more from 2020 to 2023 (APC, −16.26%; 95% CI, −37.35 to 11.95%) (Figure 2B).

Because of missingness in death counts, temporal trend lines for Hispanic and NH Other adults could not be accurately plotted. Nevertheless, absolute deaths changed over the study period. Hispanic and NH Other groups both showed measurable variations in the number of deaths from 1999 to 2023 as reported in Table 1.

### 3.5 Census region-stratified analysis

Across the four census regions, pronounced disparities were evident but narrowed over time. In 1999, the Midwest recorded the highest AAMR, followed by the Northeast, West, and South. By 2023, regional rates had converged modestly, with the Midwest retaining the highest burden and the West, South, and Northeast clustered at lower levels (Table 1).

Joinpoint analysis revealed region-specific trajectories, each characterized by inflection points in the mid-2010s. In the Midwest, mortality rates increased during 1999–2012 (APC, +4.57%; 95% CI, 0.44 to 8.86%), rose sharply but non-significantly from 2012 to 2015 (APC, +26.09%; 95% CI, −30.14 to 124.75%), continued to increase significantly between 2015 and 2020 (APC, +17.82%; 95% CI, 4.35 to 33.12%), and subsequently declined non-significantly from 2020 to 2023 (APC, −11.57%; 95% CI, −31.75 to 14.39%). The Northeast moved from an initial decline to a transient rebound and then moderated (1999–2009, −2.59%; 2009–2012, +37.63%; 2012–2015, −26.03%; 2015–2020, +12.76%; 2020–2023, −4.13%). The South demonstrated a biphasic pattern: a significant increase from 1999 to 2012 (APC, +5.26%; 95% CI, 1.78 to 8.87%), a non-significant dip in 2012–2015 (APC, −19.22%; 95% CI, −46.01 to 21.11%), and a pronounced, significant rise from 2015 to 2023 (APC, +12.12%; 95% CI, 7.73 to 16.70%). The West followed a similar arc, with an early increase (1999–2009: APC, +4.79%; 95% CI, 0.01 to 9.80%), a non-significant surge (2009–2012: APC, +29.90%; 95% CI, −10.30 to 87.34%), a non-significant decline (2012–2015: APC, −27.84%; 95% CI, −54.12 to 10.49%), and a significant sustained increase from 2015 to 2023 (APC, +8.89%; 95% CI, 4.06 to 13.94%) (Figure 2C).

### 3.6 Urbanization-stratified analysis

Urban–rural disparities in AAMR were evident throughout the study period but narrowed over time. Metropolitan areas maintained lower AAMR than nonmetropolitan areas throughout, with both showing segmented shifts that aligned around the mid-2010s (Figure 2D; Table 1). In metropolitan counties, rates rose briefly at the outset (1999–2001: APC, +25.78%; 95% CI, −10.06% to +75.92%), stabilized to mild decline (2001–2009: APC, −2.36%; 95% CI, −5.89% to +1.29%), accelerated during 2009–2012 (APC, +33.56%; 95% CI, +4.38% to +70.89%), then fell in 2012–2015 (APC, −30.40%; 95% CI, −47.10% to −8.44%), before increasing again from 2015 to 2020 (APC, +15.73%; 95% CI, +10.24% to +21.49%). Nonmetropolitan counties followed a trajectory characterized by a higher absolute burden. Rates rose significantly from 1999 to 2006 (APC, +7.42%; 95% CI, 1.28 to 13.93%), dipped non-significantly between 2006 and 2009 (APC, −13.51%; 95% CI, −41.89 to 28.74%), surged non-significantly during 2009–2012 (APC, +38.02%; 95% CI, −2.42 to 95.23%), declined non-significantly in 2012–2015 (APC, −31.46%; 95% CI, −55.55 to 5.70%), and increased significantly from 2015 to 2020 (APC, +14.00%; 95% CI, 5.06 to 23.70%).

### 3.7 Divergent mortality trends: compared with CKD overall

**Figure 3:**
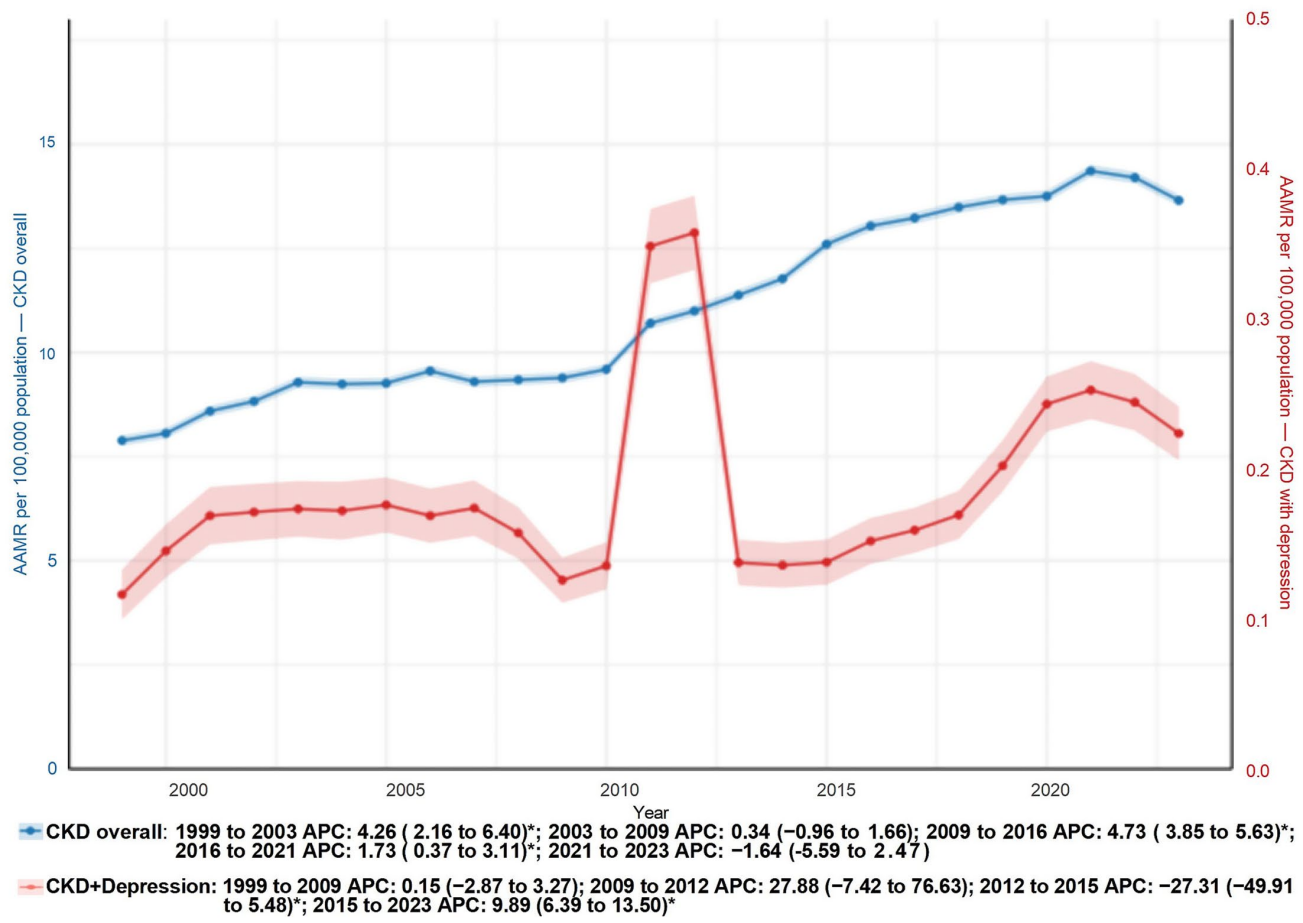
Comparative mortality trends of chronic kidney disease (CKD) overall and CKD with co-occurring depression, United States, 1999–2023. Age-adjusted mortality rates (AAMR) per 100,000 population are plotted annually from 1999 to 2023 for CKD overall (blue line, left Y-axis) and CKD with co-occurring depression (red line, right Y-axis). Shaded bands represent 95% confidence intervals. Joinpoint regression was used to identify statistically significant changes in temporal trends; fitted segments are superimposed on observed data. Annual percent change (APC) estimates for each segment are displayed in the figure legend, with asterisks denoting statistical significance (*p < 0.05). The left y-axis scale ranges from 0 to 18 per 100,000 population; the right y-axis scale ranges from 0 to 0.5 per 100,000 population.

CKD overall AAMR increased from 7.88 per 100,000 in 1999 to 13.65 in 2023 (AAPC 2.38%; 95% CI 1.75 to 3.01; *p* < 0.001), with joinpoints indicating acceleration in 1999–2003 (APC 4.26%; *p* < 0.01) and 2009–2016 (APC 4.73%; *p* < 0.001), followed by attenuation in 2016–2021 (APC 1.73%; *p* = 0.017) and a non-significant decline in 2021–2023 (APC -1.64%; *p* = 0.39) (Supplementary Table S1) In contrast, CKD-Depression maintained low absolute rates but showed a pronounced recent increase (2015–2023 APC 9.89%; 95% CI 6.39–13.50; *p* < 0.001), while its overall AAPC was not significant (2.32%; 95% CI, −3.48 to 8.47; *p* = 0.44) (Figure 3).

To formally assess whether the recent mortality trajectories of CKD overall and CKD with co-occurring depression differed in a statistically significant manner, we fitted a Poisson log linear regression model to the most recent interval of shared observation (2016–2021). The model included an offset for the natural logarithm of the annual population at risk and a year by group interaction term. In this analysis, the estimated annual percent change for CKD with depression was 12.8% (95% CI, 10.8 to 14.9%), whereas the corresponding estimate for CKD overall was 2.6% (95% CI, 2.3 to 2.9%). The year by group interaction was highly significant (*p* for interaction < 0.0001). Assessment of overdispersion yielded a dispersion parameter of 3.74, indicating mild overdispersion; given the extremely small *p* value for the interaction term (1.25 × 10^−19^), the conclusion of significant trend heterogeneity was considered robust (Supplementary Table S2).

### 3.8 Sensitivity analysis

Concerned that the transient peak observed in 2011–2012 might reflect the 2011 National Center for Health Statistics (NCHS) revision to multiple-cause-of-death coding guidelines for mental disorders (22), we conducted a sensitivity analysis restricted to the post-stabilization period (2013–2023). Joinpoint regression of this truncated series detected no statistically significant joinpoints, and the entire 11-year interval was best characterized by a single, monotonic upward trend with an APC of 7.38% (95% CI, 5.00 to 10.41%; *p* < 0.0001) (Supplementary Table S3). The absence of a downturn in the most recent years, even amid the COVID-19 pandemic, underscores the sustained and unrelenting nature of the mortality crisis in this population.

## 4 Discussion

Previous studies have revealed the persistent and bidirectional link between chronic kidney disease (CKD) and depression, in which affective symptoms exacerbate renal outcomes and CKD-related morbidity amplifies psychiatric vulnerability (23–25). However, population-level mortality patterns for their joint occurrence have remained insufficiently characterized. In this study, we leveraged nationally representative mortality data from CDC WONDER database to comprehensively describe national trends and the burden of deaths among patients with CKD and comorbid depression from 1999 to 2023, compared these trajectories with those of CKD overall, and assessed disparities across sex, age, race and ethnicity, census region, and urbanicity. Over the 25-year study period, AAMR for CKD overall increased modestly (AAPC, 2.38%; 95% CI, 1.75 to 3.01%), with the rate of increase decelerating after 2016 and a non-significant decline observed in the most recent years. In stark contrast, mortality involving CKD with co-occurring depression, while remaining at substantially lower absolute rates, exhibited a fundamentally divergent trajectory. Although the overall AAPC for the entire interval was not statistically significant, joinpoint regression identified a critical inflection point in 2015, after which the mortality rate surged at an APC of 9.89% (95% CI, 6.39 to 13.50%; *p* < 0.001). Formal testing confirmed that the temporal slopes of the two groups were non-parallel (*p* for interaction < 0.001), with the post-2015 divergence characterized by an approximately six-fold higher rate of annual increase for CKD with depression compared with CKD overall during the corresponding period. These findings demonstrate that while CKD mortality rose over the long term and has recently plateaued, mortality among individuals with co-occurring depression has entered a period of sustained and accelerating increase since 2015, substantially outpacing the secular trend of CKD alone.

Gender disparities remained persistent throughout the study period. Although men had a higher AAMR in the early years of the study period, women surpassed men in later years, consistent with the higher prevalence of depressive symptoms and diagnosed depression among women with CKD. The post-2015 upturn was more pronounced in men, who demonstrated a steeper inflection relative to their pre-2015 baseline. This sex-specific divergence mirrored the overall pattern. Prior literatures reported that women with CKD have a higher prevalence of depressive symptoms and diagnosed depression, potentially elevating baseline mortality involving CKD with depression, whereas men often experienced more abrupt deterioration once depressive comorbidity was present, contributing to steeper recent slopes (7, 26). Depression in CKD has been under-recognized in men compared with women, and delayed detection or treatment has been associated with worse clinical outcomes, which could manifest as sharper contemporary increases when comorbidity is recorded (27). Moreover, differences between men and women in cardiovascular comorbidity, systemic inflammation, and patterns of health care use in the setting of CKD and depression may also affect mortality trends (28, 29).

Stratified analyses by race, region, and urbanicity revealed a similar pattern, which was low absolute AAMR levels overall with a post-2015 acceleration that was unevenly distributed across groups. With respect to race and ethnicity, NH White and NH Black populations displayed distinct combinations of level and slope, which was consistent with prior evidence of differential depression detection and treatment in CKD and broader cardiometabolic mortality gaps (30–32). Regionally, the South sustained the highest burden with little slowing, whereas the Northeast and West exhibited more tempered trajectories and the Midwest was intermediate, reflecting known regional gradients in CKD risk factors, care access, and social determinants (33, 34). By urbanicity, nonmetropolitan areas had higher levels and, in several instances, sharper rises after 2015 than metropolitan areas, widening the urban and rural gap in line with literature on rural health care shortages, multimorbidity clustering, and rising external and cardiometabolic mortality outside metropolitan settings (35).

Age-specific patterns also supported a general rise after 2015 with different expression across age groups. Data for younger ages were incomplete. In several years and locations, there were few or no deaths under age 55, which limited precision and may have reduced apparent trends in these strata. Among age groups with sufficient data, adults aged ≥ 85 years consistently had the highest mortality burden, with clearly elevated AAMR and a sustained rise over time. Mortality slopes steepened across age strata after 2015, with the most pronounced accelerations observed in the 65–84 and ≥85 groups, mirroring the contemporary escalation documented in other subgroups. In contrast, younger strata maintained lower absolute rates and more gradual increases, indicating that the recent acceleration was concentrated among older adults who face greater multimorbidity, functional vulnerability, and competing mortality risks. Residual instability in younger groups due to sparse counts remains possible (36–38). Collectively, subgroup results support a broad but uneven acceleration after 2015 that likely reflects differences in comorbidity profiles, care fragmentation, and contextual disadvantage (39).

Measurement features of the mortality data could also affect interpretation. The absolute AAMR for CKD with depression was likely conservative because depression was often under-recorded as a contributing cause on death certificates, which shifts estimates toward the null and lowers apparent levels despite true comorbidity (32). National coding conventions have remained broadly stable from 1999 to 2023, which supports inference on temporal trends. However, variation in recognition and documentation across subgroups may differentially affect absolute levels and slopes, thereby shaping the apparent magnitude of disparities. In particular, lower recognition of depression in men relative to women and potential differences in clinical documentation across health systems and locales could attenuate or accentuate subgroup gaps without altering their direction (40). These constraints limited conclusions about absolute burden while strengthening confidence in relative changes and joinpoint timing under a consistent framework. Accordingly, the results should be understood as reflecting recorded patterns of comorbidity, not the full population prevalence of depression in CKD. Low absolute rates should not be interpreted as protective, rather, they most likely reflect incomplete capture (8, 32). Conversely, the post-2015 acceleration may partly reflect improved recognition and documentation of depression on death certificates over time, rather than solely representing worsening pathology. However, the robustness of the trend in sensitivity analyses and its divergence from plateauing CKD overall mortality argue against surveillance bias as the sole explanation. The reported trends should therefore be interpreted as reflecting both changes in recorded comorbidity and, likely, a true increase in mortality burden.

Several mechanisms may help explain the post-2015 acceleration in mortality while staying within the limits of the data. Depression may heighten mortality risk in CKD through reduced adherence to medications and dialysis regimens, impaired self-care, and lower engagement with follow-up. Moreover, Biological pathways such as chronic inflammation and neuroendocrine dysregulation may also contribute. These channels increased vulnerability to cardiovascular events, infections, and abrupt decompensation (41–43). These systemic pressures were subsequently compounded by the disruptions of the COVID-19 pandemic after 2020, which intensified mortality risk through missed outpatient visits, health care workforce strain, and pervasive social isolation (31, 44, 45). Such stressors likely exerted disproportionate effects in populations where baseline access to care and community resources were already limited. This interpretation aligns with the steeper mortality slopes observed in the present study among men, residents of the South, and individuals residing in nonmetropolitan areas. Differences by race and ethnicity in the recognition of depression, access to mental health treatment, and the burden of competing cardiometabolic risks may further contribute to the distinct combinations of mortality level and slope documented across racial and ethnic groups (8, 20, 46).

Our findings have several important implications for public health policy and clinical practice. Depression screening should be integrated systematically and repeatedly into routine CKD care (47). Brief, validated instruments are recommended for tiered assessment. The Patient Health Questionnaire-2 (PHQ-2) can be used at hospital admission and at dialysis initiation for rapid case finding. Positive screens should be followed promptly by the Patient Health Questionnaire-9 (PHQ-9) to ascertain symptom severity, guide treatment decisions, and inform the frequency of subsequent reassessment during outpatient follow-up. When clinically indicated, the Generalized Anxiety Disorder-7 (GAD-7) can identify comorbid anxiety and the Columbia-Suicide Severity Rating Scale (C-SSRS) can assess suicide risk (48, 49). Given the pronounced disparities identified in our stratified analyses, such screening efforts should be prioritized for population subgroups that exhibited either the most rapid post-2015 accelerations or the highest absolute mortality burden. These groups include men, adults aged 85 years or older, residents of the South, nonmetropolitan populations, and race-specific strata with steeper recent slopes. When applied in a targeted way, screening can support earlier detection and timely intervention, strengthen continuity and team-based care, improve resource allocation, and likely improve outcomes at the population level.

### 4.1 Limitations

Several limitations should be acknowledged. Firstly, our reliance on death certificate data may result in under ascertainment of depression, as mental health conditions may be inconsistently reported as contributing causes of death. Second, the ecological nature of the data precludes individual level causal inference, and residual confounding by unmeasured clinical and socioeconomic factors cannot be excluded. Third, death counts for certain demographic subgroups were frequently suppressed when fewer than ten deaths occurred, which limited the precision of trend estimates and precluded formal analysis for younger age groups and smaller racial and ethnic populations. Fourth, competing mortality risks may have attenuated the observed mortality rates, particularly among older adults. Individuals with CKD and depression who died from competing causes such as cardiovascular events may not have had depression recorded as a contributing cause of death, leading to potential underestimation of the true mortality burden in the oldest age groups. Finally, changes in diagnostic coding practices over the study period may have affected temporal trends.

## 5 Conclusion

In this nationally representative study of mortality trends from 1999 to 2023, individuals with CKD and co-occurring depression experienced a sustained and accelerating increase in AAMR beginning in 2015, diverging sharply from the plateauing trajectory observed for CKD overall. This post-2015 surge was particularly pronounced among men, older adults, residents of the South, and nonmetropolitan populations. These findings underscore the urgent need for systematic integration of depression screening and collaborative mental health care into routine nephrology practice, especially for populations at highest risk.

## Data Availability

The original contributions presented in the study are included in the article/Supplementary material, further inquiries can be directed to the corresponding author.
