# Supplementary material for: Temporal trends and disparities in mortality among US adults with chronic kidney disease and comorbid depression: a population-based analysis, 1999–2023
Source: Front Public Health. 2026 Jun 3;14:1860838. doi: 10.3389/fpubh.2026.1860838 (PMC13272087; doi:10.3389/fpubh.2026.1860838)
Supplement: Supplementary file 1 [file Table_1.docx]

**Supplementary Material**

| Year | Deaths | AAMR | Lower | Upper | SE |
| --- | --- | --- | --- | --- | --- |
| 1999 | 13933 | 7.88 | 7.75 | 8.02 | 0.067 |
| 2000 | 14420 | 8.06 | 7.93 | 8.19 | 0.067 |
| 2001 | 15631 | 8.59 | 8.46 | 8.73 | 0.069 |
| 2002 | 16327 | 8.83 | 8.69 | 8.96 | 0.069 |
| 2003 | 17402 | 9.28 | 9.15 | 9.42 | 0.070 |
| 2004 | 17579 | 9.24 | 9.10 | 9.38 | 0.070 |
| 2005 | 17999 | 9.26 | 9.13 | 9.40 | 0.069 |
| 2006 | 18903 | 9.55 | 9.42 | 9.69 | 0.070 |
| 2007 | 18737 | 9.30 | 9.16 | 9.43 | 0.068 |
| 2008 | 19240 | 9.34 | 9.21 | 9.48 | 0.068 |
| 2009 | 19656 | 9.39 | 9.26 | 9.52 | 0.067 |
| 2010 | 20461 | 9.60 | 9.46 | 9.73 | 0.068 |
| 2011 | 23503 | 10.70 | 10.57 | 10.84 | 0.070 |
| 2012 | 24731 | 11.00 | 10.86 | 11.14 | 0.071 |
| 2013 | 26190 | 11.38 | 11.24 | 11.52 | 0.071 |
| 2014 | 27796 | 11.77 | 11.63 | 11.91 | 0.071 |
| 2015 | 30324 | 12.60 | 12.46 | 12.74 | 0.073 |
| 2016 | 32086 | 13.04 | 12.89 | 13.18 | 0.074 |
| 2017 | 33289 | 13.23 | 13.09 | 13.37 | 0.073 |
| 2018 | 34701 | 13.49 | 13.34 | 13.63 | 0.073 |
| 2019 | 35912 | 13.67 | 13.52 | 13.81 | 0.073 |
| 2020 | 36826 | 13.75 | 13.61 | 13.90 | 0.073 |
| 2021 | 37267 | 14.36 | 14.21 | 14.51 | 0.075 |
| 2022 | 38623 | 14.20 | 14.06 | 14.34 | 0.073 |
| 2023 | 37401 | 13.65 | 13.51 | 13.79 | 0.071 |

Table S1: Deaths and AAMR of CKD overall in the United States from 1999 to 2023. (AAMR, age-adjusted mortality rates; SE, standard error.)

| Parameter | Coefficient (β) | Standard Error | z-value | p-value | Derived APC (95% CI) |
| --- | --- | --- | --- | --- | --- |
| Intercept | -60.71 | 2.581 | -23.52 | <0.0001 | — |
| Year  (CKD Overall slope) | 0.0257 | 0.00128 | 20.09 | <0.0001 | 2.6% (2.3%-2.9%) |
| Group  (CKD + Depression) | -195.5 | 21.11 | -9.26 | <0.0001 | — |
| Year × Group interaction | 0.0948 | 0.0105 | 9.06 | <0.0001 | — |
| CKD + Depression slope (sum) | 0.1205 | — | — | — | 12.8% (10.8%-14.9%) |

Table S2: Poisson Log-Linear Regression Analysis of Mortality Trend Heterogeneity Between CKD Overall and CKD with Co-occurring Depression, United States, 2016-2021. Notes: The Poisson log-linear regression model included an offset for the natural logarithm of the annual population at risk. The dependent variable was the annual death count. The model was specified as log(Deaths) = β₀ + β₁(Year) + β₂(Group) + β₃(Year × Group) + offset(log(Population)), where Group was coded as 0 for CKD Overall and 1 for CKD with co-occurring depression. The coefficient for the Year × Group interaction tests the null hypothesis that the temporal slopes of the two groups are equal. The APC for CKD Overall was calculated as (exp(β₁) – 1) × 100%; the APC for CKD with co-occurring depression was calculated as (exp(β₁ + β₃) – 1) × 100%. The 95% confidence intervals for APC estimates were derived via the delta method. Model fit statistics: null deviance = 262,467.2 on 11 df; residual deviance = 29.9 on 8 df; dispersion parameter = 3.74; AIC = 160.4. The dispersion parameter (residual deviance/residual df) suggested mild overdispersion; however, the extremely small interaction P value (1.25 × 10^-19^) renders the conclusion robust to potential variance inflation. (APC, annual percent change; CI, confidence interval; CKD, chronic kidney disease.)

| Analysis | Period | Joinpoints | APC (95% CI) | p-value |
| --- | --- | --- | --- | --- |
| Primary analysis | 2015-2023 | — | 9.89% (6.39%-13.50%) | < 0.001 |
| Sensitivity analysis (post-coding stabilization) | 2013-2023 | 0 | 7.38% (5.00%-10.41%) | < 0.0001 |

Table S3: Sensitivity Analysis: Joinpoint Regression Restricted to 2013-2023. Notes: This sensitivity analysis was restricted to the period 2013–2023 to exclude potential artifacts associated with the 2011 National Center for Health Statistics revision to multiple-cause-of-death coding guidelines for mental disorders. Joinpoint regression was performed using age-adjusted mortality rates per 100,000 population. The permutation test selected a model with zero joinpoints, indicating that the entire 11-year interval was best characterized by a single, monotonic upward trend. The APC reflects the average annual rate of change over the full restricted period. (APC, annual percent change; CI, confidence interval; CKD, chronic kidney disease.)

| Parameter | Setting |
| --- | --- |
| Software | Joinpoint Desktop, Version 5.1.0.0 (National Cancer Institute, Bethesda, MD) |
| Maximum number of joinpoints | 3 |
| Model selection method | Permutation test (Monte Carlo) |
| Number of permutations | 4,499 |
| Overall significance level | α = 0.05 |
| Minimum segment length | 3 data points (years) |
| Autocorrelated errors | Uncorrected (default) |
| Rate standard error | Based on 95% confidence intervals derived from the Poisson distribution |
| Standard population for AAMR | 2000 U.S. Standard Million Population |

Table S4: Parameter Settings for Joinpoint Regression Analysis. **Notes:** The same parameter settings were applied to all joinpoint analyses presented in this study, including (1) overall CKD-depression mortality trends, (2) subgroup-stratified analyses by sex, age, race and ethnicity, census region, and urban–rural residence, (3) comparative analysis of CKD overall versus CKD with co-occurring depression, and (4) the sensitivity analysis restricted to the 2013-2023 period. The permutation test determines the optimal number of joinpoints by comparing the fit of models with different numbers of joinpoints, with 4,499 random permutations used to assess statistical significance. The minimum segment length of three data points ensures that each identified segment contains a sufficient number of observations to produce interpretable APC estimates. Autocorrelated errors were not modeled, consistent with default Joinpoint software settings for annual mortality data. The 95% confidence intervals for AAMRs, which served as input for the joinpoint analysis, were calculated under the Poisson distribution assumption. (AAMR, age-adjusted mortality rate; APC, annual percent change; AAPC, average annual percent change.)
